# A Note on Theridion

**Published:** 1888-06

**Authors:** 


### A NOTE ON THERIDION.

In cases of scrofulosis, where the best chosen medicines do nothing, I always interpolate a dose of Theridion, which must act for eight days, and I have seen the most surprising results from it, particularly in caries and necrosis. For phthisis florida Theridion is indispensable, and effects an entire cure if given in the beginning of the disease.

In cases of rachitis, caries, and necrosis, I depend chiefly on Theridion, which, although it does not seem to affect the external scrofulous symptoms, apparently goes to the root of the evil and effectually destroys the cause of the disease. DR. BARUCH.

